# Feline Mammary Carcinoma as a Comparative Model of Human Breast Cancer: An Integrative Review

**DOI:** 10.3390/cancers18183051

**Published:** 2026-09-20

**Authors:** Carmen G. Pérez-Santana, Sara E. Cazorla-Rivero, Ruth Henríquez-Cabrera, Bernardino Clavo, Francisco Rodríguez-Esparragón

**Affiliations:** 1 Instituto Universitario de Sanidad Animal y Seguridad Alimentaria (IUSA), Universidad de Las Palmas de Gran Canaria (ULPGC), 35400 Arucas, Las Palmas, Spain; saraecari1990@gmail.com (S.E.C.-R.); hencaruth@gmail.com (R.H.-C.); afrodesp@gmail.com (F.R.-E.); 2 Unidad de Investigación Hospital Universitario de Gran Canaria Dr. Negrín, 35010 Las Palmas de Gran Canaria, Las Palmas, Spain; bernardinoclavo@gmail.com; 3 Instituto de Investigación Sanitaria de Canarias (IISC), 35019 Las Palmas de Gran Canaria, Las Palmas, Spain; 4 Facultad de Ciencias de La Salud, Universidad del Atlántico Medio, Ctra. de Quilmes, 37, Tafira Baja, 35017 Las Palmas de Gran Canaria, Spain; 5 Instituto Universitario de Enfermedades Tropicales y Salud Pública de Canarias, Universidad de La Laguna, 38296 La Laguna, Tenerife, Spain; 6 CIBER de Enfermedades Infecciosas, Instituto de Salud Carlos III, 28029 Madrid, Spain; 7 Chronic Pain Unit, Hospital Universitario de Gran Canaria Dr. Negrín, 35019 Las Palmas de Gran Canaria, Spain; 8 Radiation Oncology Department, Hospital Universitario de Gran Canaria Dr. Negrín, 35019 Las Palmas de Gran Canaria, Spain; 9 Molecular and Translational Pharmacology Group, University Institute for Research in Biomedicine and Health (iUIBS), Universidad de Las Palmas de Gran Canaria, 35016 Las Palmas de Gran Canaria, Spain; 10 Spanish Group of Clinical Research in Radiation Oncology (GICOR), 28290 Madrid, Spain

**Keywords:** feline mammary carcinoma, comparative oncology, triple-negative breast cancer, immunotherapy, proteomics, biomarkers

## Abstract

Background: Feline mammary carcinoma (FMC) is an aggressive neoplasm in cats and has attracted interest in comparative oncology because selected clinicopathological and molecular features overlap with human breast cancer. Methods: This integrative review synthesized literature was identified in PubMed/MEDLINE, Scopus, and Web of Science from database inception to 30 March 2026, focusing on epidemiology, pathology, biomarkers, tumor microenvironment, omics, therapy, and comparative oncology. Feline-derived evidence was distinguished from the human breast cancer literature, and findings were synthesized qualitatively because of substantial methodological and clinical heterogeneity. Results: FMC shows recurrent associations with aggressive histological features, hormone receptor-negative phenotypes, HER2-related signaling, immune checkpoint pathways, and emerging molecular and liquid-biopsy biomarkers. However, differences in molecular classification, methodology, treatment, cohort size, and clinical validation limit direct cross-species inference. Conclusions: FMC may serve as a complementary, question-specific comparative oncology system for studying selected aspects of aggressive mammary tumor biology, metastasis, tumor–host interactions, biomarkers, and therapeutic response. Its translational relevance remains hypothesis-generating and requires standardized methods and independent clinical validation.

## Simple Summary

Feline mammary carcinoma (FMC) is one of the most aggressive cancers in cats, characterized by rapid progression, a high risk of metastasis, and poor prognosis. This tumor shares important clinical, histopathological, and molecular similarities with human breast cancer, particularly with aggressive subtypes such as triple-negative and HER2-positive breast cancer, supporting its potential utility for selected comparative oncology questions. In this review, we summarize current knowledge of FMC, focusing on its epidemiology, pathological features, prognostic factors, molecular alterations, tumor microenvironment, and emerging therapeutic strategies. Recent advances in molecular and multi-omic approaches have identified common pathways involved in tumor growth, immune regulation, epithelial-to-mesenchymal transition, and metastasis in feline and human mammary tumors. Although surgery remains the cornerstone of treatment in cats, the identification of new biomarkers and the development of targeted and immunotherapeutic approaches may improve disease management and provide further opportunities for translational research. Overall, FMC may contribute to comparative studies of aggressive mammary cancer while simultaneously advancing feline cancer diagnosis and treatment.

## 1. Introduction

Feline mammary neoplasms represent one of the most significant oncological conditions in domestic cats, ranking as the third most common tumor type after hematopoietic and cutaneous neoplasms [1,2,3]. Their prevalence varies geographically, primarily due to differences in spaying practices, but they are universally recognized for their aggressive biological behavior, high metastatic potential, and the remarkably high proportion of malignant cases, estimated at 85–95% [4,5].

Feline mammary carcinoma (FMC) is the most common malignant mammary tumor in cats and has been investigated as a spontaneous comparative model of human breast cancer because of its histological, molecular, and clinical similarities [1,2,3]. Accounting for over 80% of feline mammary neoplasms, FMCs demonstrate an infiltrative growth pattern, early lymphatic spread, and poor clinical outcomes [4,5,6].

Histologically, FMC presents a diverse spectrum of subtypes, including tubulopapillary, solid, cribriform, and micropapillary patterns. These are typically defined by pronounced nuclear pleomorphism, lymphovascular invasion, and high mitotic indices [7,8,9,10]. Notably, several subtypes identified in human breast cancer, such as ductal carcinoma in situ, as well as tubular, papillary, cribriform, invasive micropapillary, and secretory carcinomas, are also documented in cats [5,7,11]. Among these, the cribriform and invasive micropapillary variants are particularly aggressive, sharing distinct clinicopathological features with their human counterparts [5,12].

With a malignancy rate of approximately 85%, comparable to canine figures but significantly exceeding the 15% reported in women, FMC may provide a useful comparative oncology model [5,11,13]. Its comparative interest is supported by selected epidemiological, biological, and molecular similarities to human breast cancer [5,10,12]. These similarities have been investigated particularly in hormone receptor-negative/triple-negative and HER2-associated phenotypes [10,14,15]. Despite advancements in surgical and chemotherapeutic protocols, the prognosis remains guarded; median survival times rarely surpass one-year post-mastectomy [5,7]. Consequently, the limited and heterogeneous evidence for systemic therapies has encouraged investigation of targeted and immune-related pathways [16,17,18,19,20,21].

The aim of this integrative review is not to establish feline mammary carcinoma as a direct surrogate of human breast cancer, but to critically evaluate the extent of clinicopathological, molecular, immunological, and therapeutic convergence between the two diseases and to identify the specific areas in which feline tumors may provide comparative value.

### 1.1. Review Design and Literature Search

This study was designed as an integrative review of the literature on feline mammary carcinoma (FMC), with emphasis on epidemiology, clinicopathological characteristics, prognostic and predictive biomarkers, molecular and multi-omic features, tumor microenvironment, therapeutic approaches, and comparative oncology. Search was conducted in PubMed/MEDLINE, Scopus, and Web of Science Core Collection from database inception to 30 March 2026. Given the integrative nature of this review, the search process was structured and iterative, combining core terms related to feline mammary carcinoma with topic-specific terms representing the main domains addressed in the review, including comparative oncology, human breast cancer, molecular phenotypes, biomarkers, tumor microenvironment, immunotherapeutic approaches, proteomics, phosphoproteomics, peptidomics, and tyrosine kinase inhibitors. Database-specific electronic search strategies, including search fields, Boolean operators, and applied limits, are reported in Supplementary Table S1. The recent literature was prioritized to provide an updated overview, while older publications were retained when they provided foundational epidemiological, pathological, molecular, or clinical evidence. Reference lists of relevant publications and reviews were manually screened to identify additional pertinent studies. Because the review was designed as an integrative rather than a systematic review, the search and selection process was iterative and no quantitative record-by-record screening log was maintained; therefore, retrospective estimates of records identified or excluded were not generated.

### 1.2. Eligibility Criteria

Studies were considered eligible when they addressed FMC or feline mammary neoplasia in relation to epidemiology, reproductive risk factors, histopathological classification or grading, clinical staging, prognostic or predictive factors, immunohistochemical characterization, molecular subtyping, epithelial–mesenchymal transition, tumor microenvironment, immune regulation, omics-based approaches, emerging biomarkers, surgery, systemic treatment, targeted therapy, immunotherapy, or comparative oncology. Peer-reviewed original studies, clinical studies, pathological and immunohistochemical investigations, molecular and multi-omic studies, and relevant comparative studies were included. Review articles were used for historical or conceptual context and to identify primary studies, with preference given to primary feline evidence when available. Human breast cancer publications were included selectively to define the corresponding human framework and were not treated as direct evidence of feline disease. Conference abstracts, editorials, commentaries, studies focused on unrelated neoplasms without a relevant comparative component, and publications with insufficient information were excluded. Isolated case reports were generally excluded unless they provided particularly relevant evidence on rare entities, biomarkers, or therapeutic observations.

### 1.3. Study Selection and Evidence Synthesis

Study selection was guided by the objectives and thematic scope of the review. Titles and abstracts were assessed for relevance, followed, when appropriate, by full-text evaluation. Additional studies identified through reference-list screening were considered when they provided relevant evidence not adequately represented in the database searches. Selection was based on scientific relevance, contribution to the predefined thematic domains, and adequacy of the available evidence rather than on a predefined publication quota. Data extracted included study characteristics, feline population or biological material, tumor classification, histopathological features, clinical stage, prognostic variables, immunohistochemical markers, molecular alterations, immune and tumor-microenvironment characteristics, omics findings, candidate biomarkers, therapeutic interventions, clinical outcomes, and comparative observations relevant to human breast cancer. Evidence was synthesized qualitatively because of substantial heterogeneity in study design, sample size, pathological classification, immunohistochemical methodology, molecular techniques, treatment protocols, and clinical endpoints. Feline-derived findings were explicitly distinguished from observations derived from human oncology, and phenotypic or pathway-level similarities were interpreted as comparative or hypothesis-generating evidence rather than as evidence of biological equivalence. No quantitative meta-analysis was performed.

## 2. Epidemiology and Risk Factors

Feline mammary carcinoma stands as one of the most prevalent and biologically aggressive malignancies in the feline population, representing 12–17% of all diagnosed tumors [1,2,3]. Global epidemiological surveys consistently place FMC among the three most common neoplasms in this species [1,2]. Its clinical profile is characterized by invasive growth, frequent lymphatic and distant dissemination, and poor outcome [5,11,12].

A comprehensive retrospective analysis by Souza et al. (2024), involving 418 cats and 858 lesions, underscores the severity of this condition; the study found that 88.8% of mammary lesions were malignant, with nearly half of these cases demonstrating lymph node metastasis at the time of diagnosis [11]. Furthermore, this research expanded the known pathological spectrum of the disease by documenting rare or previously unreported entities, such as mammary sporotrichosis, benign phyllodes tumors, and basaloid carcinoma [11].

### 2.1. Hormonal and Reproductive Risk Factors

The primary epidemiological driver for FMC development is prolonged exposure to ovarian hormones. Intact females face a seven-fold higher risk of developing mammary neoplasia compared to spayed individuals [2,4,22]. While early ovariohysterectomy provides a significant protective effect, delayed sterilization or the administration of exogenous progestins substantially elevates susceptibility [2,4,5]. Nevertheless, the occurrence of FMC in ovariectomized cats suggests that non-hormonal oncogenic pathways and genetic predispositions also play a critical role in tumorigenesis [4].

In both species, the disease is linked to endocrine disturbances, such as gonadal dysfunction or exposure to exogenous hormones. For instance, the administration of cyproterone acetate in males has been directly associated with the onset of mammary carcinoma [13].

Clinical data regarding male cats indicate a highly aggressive disease course. Despite a median survival of approximately 344 days, many cases present with advanced prognostic markers, including tumors exceeding 3 cm and evidence of lymphatic invasion [23]. The terminal stages often involve pulmonary metastasis, manifesting as respiratory distress and cachexia. These findings highlight the need for careful risk-benefit assessments when using progestin-based therapies, such as medroxyprogesterone acetate, and emphasize the necessity of long-term clinical surveillance for both sexes to facilitate early detection [24].

### 2.2. Age, Sex, and Breed Distribution

FMC predominantly affects middle-aged to geriatric cats, with a peak incidence between 10 and 12 years of age. This timing reflects the cumulative impact of hormonal exposure and the gradual accrual of genetic alterations [1,5]. Although the disease is most common in mixed-breed feline populations, which constitute the majority of the global domestic population, a higher risk has been suggested in Siamese cats [2,5]. However, this potential breed predisposition remains a subject of debate, as it may be influenced by regional population structures rather than underlying genetic susceptibility.

In stark contrast, mammary neoplasms in male cats are exceptionally rare, accounting for only 1% of all cases [23]. Diagnosed at a slightly older mean age of 12.8 years, male FMC offers a compelling parallel to male breast cancer in humans [23,24].

## 3. Histopathology and Staging

### 3.1. Overview and Rationale for Updated Staging Approaches

In feline oncology, prognostic evaluation relies heavily on clinical staging to dictate therapeutic strategies and predict survival. While the traditional World Health Organization (WHO) tumor–node–metastasis (TNM) classification is widely utilized, it presents significant limitations when applied to feline mammary tumors (FMTs) [25,26,27]. A primary deficiency is the system’s failure to differentiate between large primary tumors and those with regional metastasis; both may fall within Stage III despite distinct prognostic implications [25,28]. Furthermore, the absence of a T4 category; used in human medicine to classify tumors invading the thoracic wall or skin, limits the system’s descriptive power [25].

To address these gaps, researchers proposed integrating a T4 category for ulcerated lesions [25]. Building upon this, a staging system adapted from the 7th edition of the AJCC Cancer Staging Manual was evaluated in 75 cats [28]. This modified framework subdivides Stage III into IIIA (T3N0M0), IIIB (T4N0M0), and IIIC (AnyTN1M0), aiming to refine risk stratification and align veterinary practices more closely with human oncological standards.

### 3.2. Histopathological Diversity

**Figure 1:**
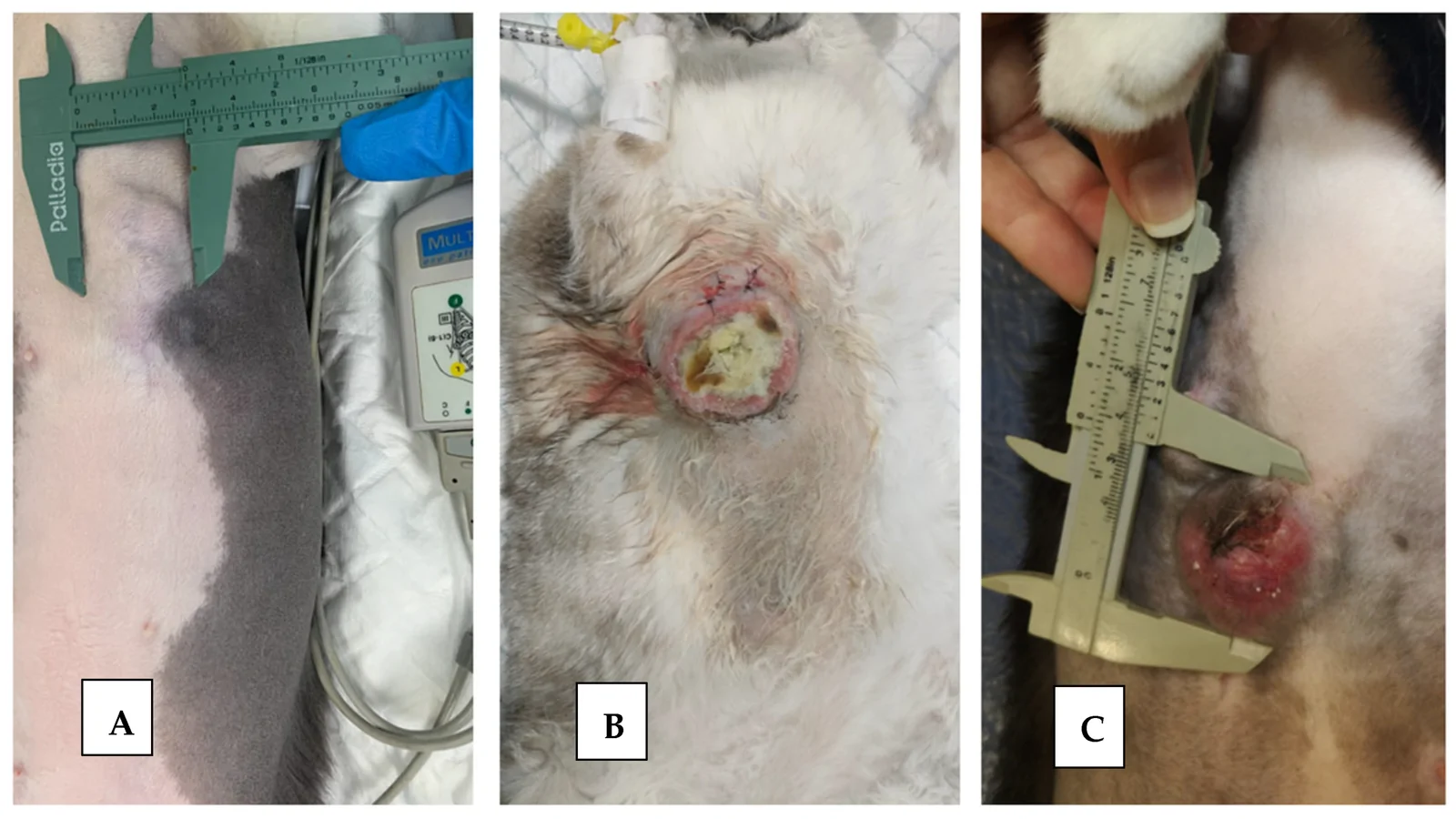
Representative clinical presentation of feline mammary carcinomas. Gross appearance of three feline mammary neoplasms with different histopathological diagnoses: (**A**) moderately differentiated tubulopapillary mammary carcinoma, Grade II; (**B**) undifferentiated adenocarcinoma, intermediate grade (Grade II); and (**C**) simple solid mammary carcinoma, Grade III.

Feline mammary tumors exhibit substantial morphological diversity that parallels the spectrum of human breast cancers (Figure 1). Documented subtypes include ductal carcinoma in situ, as well as tubular, papillary, cribriform, micropapillary, lobular-like, and secretory carcinomas [10,11,29].

Among these, cribriform and invasive micropapillary variants are especially significant due to their aggressive biological behavior and poor prognosis [9,11].

A large clinicopathological series of 418 cats identified tubulopapillary carcinoma (17.1%), cribriform carcinoma (16.8%), and malignant adenomyoepithelioma (12.1%) as the most frequent malignant histological categories [11]. Tubulopapillary and cribriform carcinomas commonly show marked anisocytosis/anisokaryosis, high mitotic counts, and necrosis, whereas p63 immunohistochemistry was useful in identifying malignant adenomyoepithelioma in the large 2024 series [11].

### 3.3. Grading Systems and Clinical Staging Integration

To ensure diagnostic consistency, several histological grading systems adapted from human medicine are applied to FMTs. The Elston and Ellis (EE) or Nottingham system evaluates tubular formation, nuclear pleomorphism, and mitotic count to categorize tumors into Grades I, II, or III (Table 1). Given the high mitotic rates in cats, the Mitotic-Modified Elston and Ellis (MMEE) and Revised Elston and Ellis (REE) systems provide feline-specific adaptations, with the REE system additionally incorporating nuclear form and lymphovascular invasion [7,8,30]. Histological grade remains a powerful prognosticator; for instance, Grade III carcinomas are associated with a nearly tenfold increase in mortality risk compared to Grade I [7].

These grading schemes are complemented by morphological classifications (Table 2) and clinical staging (Table 3), which integrates tumor size, nodal status, and distant metastasis [25,26,27,29]. The proposed revised staging system was designed to refine prognostic stratification by distinguishing subgroups within Stage III. Specifically, Stage IIIC cases (nodal metastasis) carry the worst prognosis, with a median overall survival of only 288 days [28]. Additionally, skin ulceration (present in 18.7% of cases) is an independent negative prognostic factor, reducing median survival to approximately nine months [25]. This mirrors human oncology, where ulceration is a formal component of staging frameworks.

**Figure 2:**
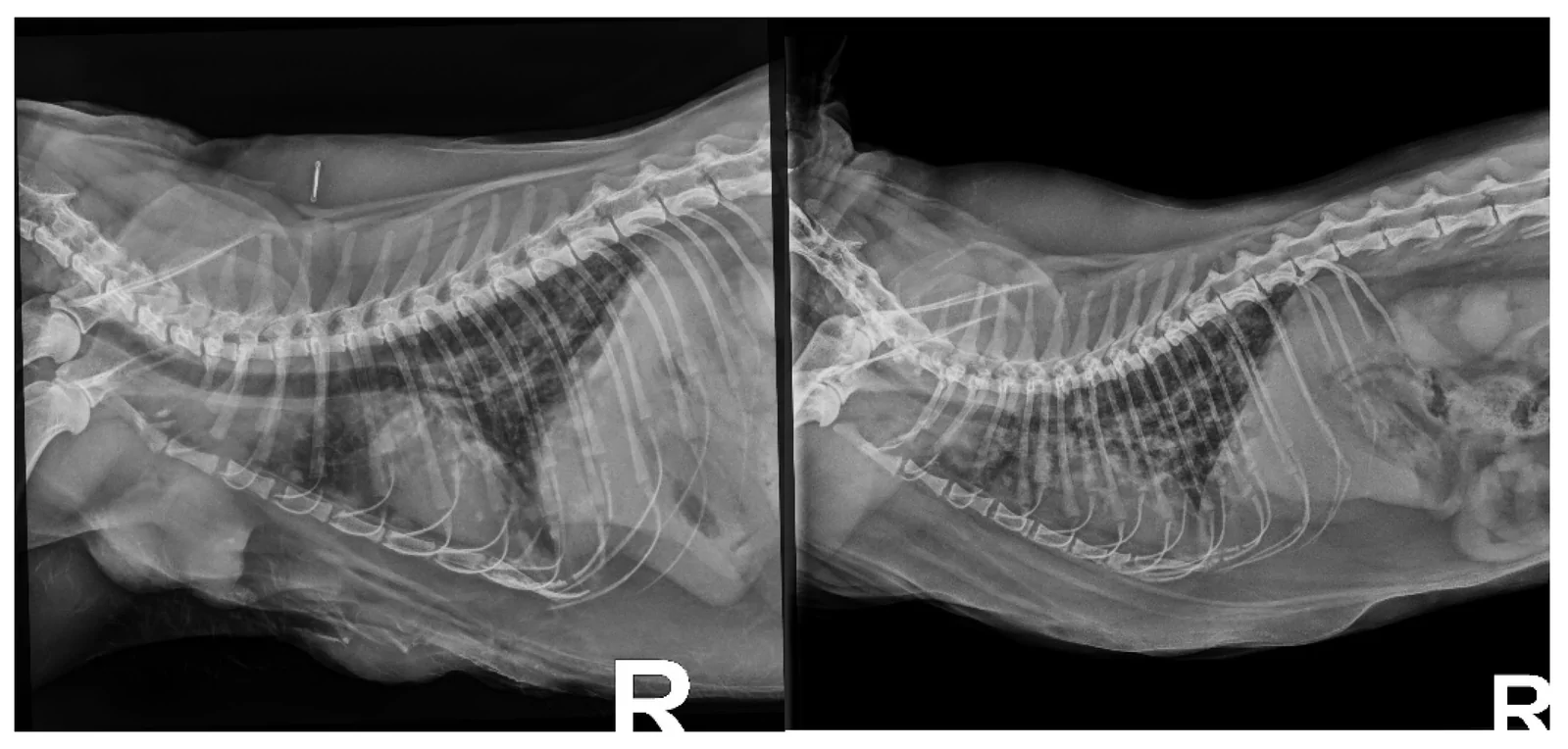
Thoracic radiographic findings consistent with pulmonary metastatic disease in a cat with feline mammary carcinoma.

Thoracic imaging plays a central role in the detection of distant metastatic disease in cats with mammary carcinoma, particularly pulmonary involvement (Figure 2), and therefore represents an essential component of clinical staging.

## 4. Comparative Clinicopathological Insights

Recent investigations have refined the understanding of the clinical behavior and pathological spectrum of feline mammary lesions by focusing on the critical differentiation between neoplastic and non-neoplastic conditions.

### 4.1. Age-Related and Demographic Correlations

A clear correlation exists between age and the biological nature of mammary lesions because cats diagnosed with malignant tumors are significantly older than those presenting with benign tumors or non-neoplastic conditions [2,5,11]. While crossbred cats predominate in these cohorts and reflect global population demographics, the persistent overrepresentation of older and intact females reinforces the established link between cumulative hormonal exposure and malignancy [1,4,11].

### 4.2. The Non-Neoplastic and Benign Spectrum

Recognizing non-neoplastic conditions is essential for accurate differential diagnosis as these processes often mimic the clinical appearance of malignancy. Mastitis, usual ductal hyperplasia, and fibroadenomatous hyperplasia represent the most frequent non-neoplastic findings, while adenomas and ductal papillomas remain the most prevalent benign neoplasms [11]. Notably, the strong association observed between usual ductal hyperplasia and concurrent malignancy in more than half of reported cases suggests a potential preneoplastic role that mirrors observations in human breast pathology.

### 4.3. Emerging Pathological Entities and Rare Variants

Recent diagnostic efforts have expanded the feline mammary landscape by documenting rare entities previously recognized primarily in human or canine oncology. These include basaloid carcinoma and benign phyllodes tumors along with mammary sporotrichosis, which is a significant finding in younger cats that highlights how infectious granulomatous processes can clinically present with nodal involvement and lead to a misdiagnosis of metastatic neoplasia [11].

Furthermore, detailed microscopic comparisons reveal that while high-grade carcinomas such as tubulopapillary and cribriform variants consistently exhibit high mitotic indices and necrotic foci, malignant adenomyoepitheliomas often present with lower cytological atypia and more indolent behavior [7,11]. Although these findings suggest that adenomyoepitheliomas may carry a more favorable prognosis than other feline mammary carcinomas, further prospective research is required to confirm their specific clinical trajectory.

Feline mammary carcinoma exhibits marked molecular, histopathological, and immunological heterogeneity, which significantly influences both prognosis and therapeutic selection.

### 4.4. Molecular Subtypes and Immunohistochemical Profiling

Immunohistochemical profiling reveals that feline tumors frequently display variable protein expression of the estrogen (ER) and progesterone (PR) steroid receptors, generally at lower levels than those observed in human luminal breast cancers. The HER2 (ERBB2) receptor tyrosine kinase, which promotes cell growth, is overexpressed in approximately 10–20% of cases and is typically associated with high histological grade and elevated proliferative activity [14,15,16]. Notably, a substantial proportion of FMCs lack ER, PR, and HER2 expression. These triple-negative subtypes exhibit the most aggressive biological behavior, paralleling the clinical trajectory of triple-negative breast cancer in human patients [14,16].

Beyond receptor status, several markers serve as vital prognostic indicators. High proliferation indices assessed via Ki-67 correlate with aggressive clinicopathological features and shorter survival, with a 14% cutoff proposed in one major study [31]. Reduced E-cadherin expression has also been associated with malignant tumors and metastases, supporting its investigation as a marker of invasive behavior [32].

## 5. Immunohistochemical Findings and Cellular Dynamics

Immunohistochemical analysis provides critical insights into the cellular origin and phenotypic plasticity of feline mammary tumors. Cytokeratin expression is strongly marked in neoplastic epithelial cells, whereas stromal fibroblasts exhibit vimentin immunoreactivity, reflecting their epithelial and mesenchymal phenotypes, respectively.

### 5.1. Evidence of Epithelial-to-Mesenchymal Transition (EMT)

Co-expression of cytokeratin and vimentin has been described in feline mammary carcinomas, particularly in hormone receptor-negative tumors with basal-like characteristics [10]. In human oncology, EMT is a recognized biological process associated with tumor-cell plasticity, invasion, and dissemination [33,34]. In FMC, however, marker co-expression should be interpreted as evidence of phenotypic plasticity rather than as proof of a fully conserved EMT program across species.

### 5.2. Comparative Proliferative Activity

The assessment of cellular proliferation via Ki-67, a protein strictly associated with active phases of the cell cycle, reveals important differences between primary and secondary sites. Higher Ki-67 indices have been reported in nodal and distant metastases compared with corresponding primary tumors, supporting an association between increased proliferative activity and metastatic progression [31].

While these findings align with the expected progression of malignant disease, standardized prognostic cut-off values for Ki-67 in feline mammary carcinoma remain a subject of ongoing debate [7,31,35]. Interestingly, the indices reported in recent specific cases have occasionally fallen below previously proposed thresholds, suggesting that even tumors with relatively low proliferative counts can demonstrate significant metastatic potential [31].

The tumor microenvironment (TME) is a complex ecosystem that critically shapes disease progression and therapeutic responsiveness [17]. Feline mammary carcinoma utilizes “immune checkpoints”; regulatory molecules that normally prevent autoimmunity, to evade the immune system [16,18,33,34,36,37].

Expression of PD-1 (Programmed Cell Death 1), a receptor on immune cells, and its ligand PD-L1, found on tumor cells, correlates with higher histological grades and lymphatic invasion [16]. Other identified checkpoints include CTLA-4, which contributes to immune tolerance [38]; TIM-3, a protein whose expression correlates with clinical outcomes [37]; and VISTA, a recently identified regulator of T-cell responses [18]. Furthermore, metabolic mediators like leptin and its receptor ObR connect nutritional status with tumor aggressiveness, while dysregulated inflammatory cytokines such as IL-6 and TNF-alpha further facilitate immune escape [36].

The presence of functional tumor-infiltrating lymphocytes (TILs) and active checkpoint axes supports the integration of immune-targeted strategies. For example, monoclonal antibody-based therapies targeting HER2 have shown preclinical potential. HER2-targeted approaches have shown antiproliferative effects in feline cell-based models [39], while checkpoint pathways such as PD-1/PD-L1, CTLA-4, VISTA, and TIM-3 have been characterized in FMC [16,17,18,37,38]. These findings provide a rationale for further preclinical investigation, but do not establish clinical efficacy or cross-species therapeutic equivalence.

Recent large-scale proteomic studies have identified over 16,000 proteins in FMC. Metastatic tumors showed distinct protein signatures, including increased IL18R1 and DIMT1 and alterations in proteins such as GSDMC, while NOP14 and SCN8A were among proteins showing decreased expression in metastatic/high-grade groups [40].

Metastatic progression is further supported by the enrichment of ACTN4 (a cytoskeletal remodeling protein) and GSDMC (a protein involved in inflammatory cell death or pyroptosis), while tumor-suppressive proteins like NOP14 and the SCN8A sodium channel are significantly downregulated [40]. These alterations converge with earlier findings regarding phosphoproteomics, where the dysregulation of signaling enzymes like PPP1CA and metabolic regulators like PRKAG3 reinforces FMC as a robust model for comparative research [41].

The need for minimally invasive diagnostics has prompted the exploration of liquid biopsy through serum peptidomics. Unlike proteomics, which characterizes the complete protein repertoire of a biological sample, peptidomics focuses on the identification and quantification of endogenous peptides naturally present in biological fluids and may provide highly sensitive biomarkers of disease progression and metastatic behavior. Using non-trypsinized LC-MS/MS, Paphussaro et al. (2024) identified distinct serum peptidomic signatures associated with metastatic (mFMC) and non-metastatic feline mammary carcinoma (NmFMC) [42].

The serum peptidomic study identified differential peptide/protein-associated signatures between metastatic and non-metastatic FMC, including peptides derived from proteins involved in proliferation, metabolism, apoptosis resistance, and tumor progression [42].

The metastatic group showed a distinct serum peptidomic profile that included peptides associated with pathways implicated in tumor progression and metastasis [42]. These findings support serum peptidomics as a promising biomarker-discovery approach, but they do not yet establish its value for clinical risk stratification or prediction of therapeutic response; larger cohorts and independent validation are required [42].

## 6. Therapeutic Strategies in Feline Mammary Carcinoma

**Figure 3:**
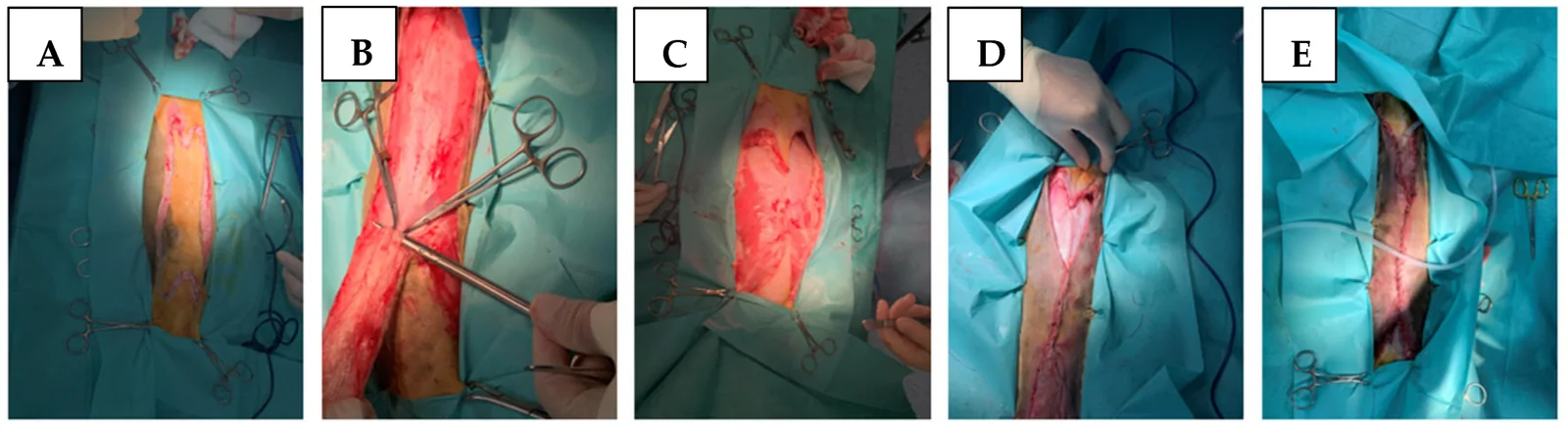
Radical bilateral mastectomy using a modified “butterfly-wing” technique and placement of a Jackson-Pratt drainage system. (**A**) Preoperative skin marking showing the bilateral incision pattern in a “butterfly-wing” configuration designed to reduce tension during primary closure; (**B**) Tissue dissection and complete excision of the bilateral mammary chain; (**C**) Surgical bed following complete resection, displaying the resulting tissue defect; (**D**) Placement of a Jackson-Pratt suction drain within the wound bed to prevent fluid accumulation and facilitate local analgesia administration; (**E**) Final skin closure using a continuous suture pattern showing adequate tension distribution.

Surgical excision remains the primary cornerstone of treatment for feline mammary tumors, as aggressive intervention is typically required to address the high rate of malignancy. Bilateral mastectomy is generally recommended because feline mammary tumors are frequently multifocal and aggressive [6,11] (Figure 3). Despite this, achieving complete surgical margins remains a persistent challenge, particularly in cases of ulcerated or infiltrative tumors where local recurrence is frequent [8,19].

Given these surgical limitations and the relative scarcity of comprehensive clinicopathological data compared to canine mammary oncology, precise histopathological classification and staging are essential for effective postoperative management [3,25,28]. Standardizing feline staging may improve prognostic assessment and facilitate more reproducible comparative studies [25,28]. Ultimately, a unified diagnostic approach is vital for overcoming the current limitations of cytotoxic therapies and improving long-term outcomes for feline patients.

### 6.1. Targeted and Comparative Therapeutic Strategies

Evidence for conventional chemotherapy in FMC remains limited and heterogeneous [19,43], encouraging investigation of targeted approaches. These strategies focus on growth factor receptors and immune regulatory pathways that shape the tumor microenvironment.

#### 6.1.1. Tyrosine Kinase Inhibitors (TKIs) and Clinical Response

Interest in TKIs is driven by the documented expression of receptors such as VEGFR (vascular endothelial growth factor receptor), PDGFR (platelet-derived growth factor receptor), and KIT in feline mammary carcinoma [20,21].

Toceranib phosphate, a multi-target TKI, has demonstrated a clinical benefit rate of 64.7% in cats with macroscopic mammary adenocarcinoma [21]. In the prospective toceranib study, objective responses were reported in a subset of cats, with a median overall survival of 145 days [21]. A notable observation in these trials is “biological discordance,” where primary tumors respond to treatment while distant metastases progress. The discordant responses observed between primary tumors and metastases highlight the potential importance of interlesional biological heterogeneity, although the mechanisms were not established by that study.

Beyond toceranib, Lapatinib, a dual inhibitor of EGFR (epidermal growth factor receptor) and HER2, has shown potent antiproliferative effects in feline cell lines [20]. However, pharmacokinetic studies indicate that cats require optimized dosing due to lower systemic exposure compared to dogs or humans.

#### 6.1.2. Comparative Advances in Immunotherapy

Modern oncology categorizes tumors as immunologically “hot” (inflamed), “excluded,” or “cold” (immune-desert), a distinction that dictates responsiveness to immunotherapy [44,45,46]. Because selected FMCs show hormone receptor-negative/triple-negative phenotypes and immune checkpoint alterations, FMC may provide a complementary system for investigating tumor–immune interactions relevant to aggressive mammary cancer [10,16,17,18].

The therapeutic landscape is currently centered on immune checkpoint inhibitors (ICI). Beyond the PD-1/PD-L1 axis, several emerging targets have been identified in both species:

- LAG-3: An inhibitory receptor co-expressed on exhausted T cells. Its blockade, often combined with PD-1 inhibitors, shows synergistic potential in reversing T-cell exhaustion [45,46].
- VISTA: An immune-regulatory checkpoint that has been investigated in aggressive feline mammary carcinoma subtypes and proposed as a potential therapeutic target [18].
- TIM-3: A regulator of innate immunity whose expression in FMC paradoxically correlates with clinical outcomes, mirroring human TNBC data [37].
- CTLA-4: A fundamental regulator of T-cell activation. Its soluble isoform has been detected in both human and feline serum, serving as a potential non-invasive biomarker [38].

### 6.2. Future Directions: Multimodal Integration

The potential integration of TKIs with immune-modulatory strategies represents a hypothesis for future FMC studies. While current studies are often limited by small sample sizes and variable protocols, the observed activity of these agents supports further investigation, but does not establish comparative efficacy [16,18,20,21,37,38].

Moving forward, larger randomized trials are essential to define the optimal role of these therapies, particularly in the adjuvant setting for microscopic disease. Harmonized diagnostic frameworks could facilitate more rigorous comparative studies and evaluation of personalized approaches, while clinical benefit in either species remains to be established [6,7].

## 7. Feline Mammary Carcinoma and Human Breast Cancer: A Comparative Oncology Perspective

The comparative interest in FMC is based on the occurrence of several clinicopathological and molecular features that overlap with those described in human breast cancer. These include aggressive invasive behavior, lymphatic and distant dissemination, alterations in hormone receptor expression, HER2-associated signaling, proliferative activity, tumor–immune interactions, and dysregulation of pathways involved in tumor progression [5,12,14,15,16,17,18]. However, these similarities should be interpreted at the level of specific biological processes rather than as evidence of complete biological or molecular equivalence. In particular, the immunohistochemical phenotypes described in FMC should not be considered direct substitutes for the molecularly defined subtypes of human breast cancer.

### 7.1. Clinicopathological Convergence

FMC is characterized by a high proportion of malignant tumors and frequent invasive growth, while tumor size, histological grade, lymphovascular invasion, and lymph-node involvement have been investigated as prognostic factors [5,7,8,25,30]. Several histological patterns have been described in feline mammary carcinomas, including tubulopapillary, solid, cribriform, and invasive micropapillary patterns [5,9]. Invasive micropapillary carcinoma is of particular comparative interest because its histological architecture and propensity for lymphatic dissemination have parallels in human breast carcinoma [9]. Nevertheless, morphological similarity alone does not establish equivalence between feline histological entities and the considerably more extensive classification used in human breast pathology.

Histological grading has been investigated as a prognostic tool in FMC, with several studies reporting associations between higher grade, increased proliferative activity, and poorer outcome [7,8,30]. However, the prognostic performance of individual grading systems has varied among studies, and differences in methodology have complicated direct comparison of published cohorts [5,7,26]. These limitations are relevant when FMC is considered from a comparative oncology perspective because reproducible pathological classification is a prerequisite for meaningful cross-species comparisons.

Tumor burden and regional lymph-node involvement also represent areas of clinicopathological convergence. Larger primary tumors and lymph-node metastasis are generally associated with more advanced disease and poorer outcome in cats [5,7,8,25,30]. Similar variables constitute established components of human breast cancer staging. Thus, these parameters may provide useful common clinical endpoints for comparative studies, although staging systems and treatment implications remain species-specific.

The spontaneous development of FMC in immunocompetent animals is a potential comparative advantage. Unlike experimentally induced tumors or conventional cell-line xenografts, naturally occurring FMC develops within an intact host and is influenced by the tumor microenvironment, immune system, stromal interactions, and naturally occurring metastatic processes [12,17]. This feature may be particularly informative for studying tumor progression and host–tumor interactions. However, spontaneous development should not be interpreted as evidence that FMC reproduces the complete biological spectrum of human breast cancer.

### 7.2. Hormone Receptors, HER2, and Phenotype-Based Molecular Classification

Immunohistochemical evaluation of estrogen receptor (ER), progesterone receptor (PR), HER2, cytokeratins, and Ki-67 has been widely used to characterize FMC [14,15,16,17,18,31,35]. Compared with human breast cancer, feline mammary carcinomas show a relatively high frequency of hormone receptor-negative tumors, particularly among aggressive carcinomas [5,12,14,15]. This feature has prompted investigation of FMC as a potential comparative system for selected hormone receptor-negative and basal-like human breast cancers [12].

Feline mammary carcinomas can be grouped using IHC-based surrogate phenotypes analogous to those applied clinically in human breast cancer. Soares et al. identified luminal A, luminal B, HER2-positive, and triple-negative/basal-like phenotypes in feline mammary carcinomas and reported differences in survival between these groups [14,15]. In particular, triple-negative/basal-like tumors were associated with shorter survival and disease-free intervals in the studied cohorts [15]. These findings support the biological heterogeneity of FMC and provide a rationale for comparative investigation of aggressive mammary tumor phenotypes.

Nevertheless, the term “triple-negative” requires particular caution in a comparative context. In human breast cancer, TNBC is defined clinically by the absence of ER, PR, and HER2 expression but represents a heterogeneous group of tumors with distinct transcriptional, genomic, and biological characteristics [47]. Therefore, an ER−/PR−/HER2− feline tumor may be regarded as a phenotypic analogue of human TNBC, but receptor negativity alone does not demonstrate molecular equivalence to any specific human TNBC subtype. Comparative gene-expression analyses have identified similarities between feline mammary carcinomas and human TNBC [48], supporting further investigation, but these observations remain insufficient to establish that FMC reproduces the full molecular heterogeneity of human TNBC.

HER2 provides another important example of both biological convergence and methodological limitation. HER2 expression has been detected in feline mammary carcinomas, and feline HER2 has been investigated at both the protein and gene levels [18,20,39]. However, reported frequencies of HER2 positivity have varied considerably between studies. Soares et al. demonstrated that HER2 assessment in FMC requires optimized IHC and in situ hybridization methodologies and emphasized the inconsistency of previously available data [49]. Similarly, Rasotto et al. detected HER2 overexpression in only 5.5% of feline mammary carcinomas using an optimized IHC protocol [49]. These findings indicate that differences in antibodies, staining protocols, scoring criteria, and analytical thresholds may substantially influence the reported prevalence of HER2-positive FMC.

This methodological issue is particularly important because HER2 testing is highly standardized in human breast cancer, where validated IHC and in situ hybridization criteria are used to determine eligibility for HER2-directed treatment [50]. Consequently, the detection of HER2 expression in feline tumors should be interpreted as evidence of a potentially conserved biological pathway rather than as direct evidence that feline HER2-positive tumors are equivalent to human HER2-positive breast cancer.

### 7.3. Proliferation, Invasion, and Metastatic Progression

Proliferative activity is another area of convergence between FMC and human breast cancer. Ki-67 expression has been investigated extensively in feline mammary tumors and has been associated with histological grade and clinical outcome in several studies [31,35]. However, reported cut-off values and the strength of prognostic associations have varied among studies, and the absence of standardized methodology remains an important limitation [5,26,31,35]. Accordingly, Ki-67 may be considered a promising prognostic marker in FMC, but feline-specific thresholds should not be directly extrapolated from human breast cancer.

The invasive and metastatic behavior of FMC provides a further opportunity for comparative investigation. Feline mammary carcinomas may exhibit lymphovascular invasion, regional lymph-node metastasis, and distant dissemination, particularly to the lungs [5,8,15,25]. The presence of invasive micropapillary morphology has also been associated with lymphatic involvement [9]. These features make naturally occurring FMC potentially informative for studying mechanisms of tumor dissemination.

At the molecular level, epithelial-to-mesenchymal transition (EMT)-associated processes have been investigated in feline mammary carcinoma [33,34,41]. The co-expression of epithelial and mesenchymal markers has been described in feline tumors, consistent with phenotypic plasticity associated with invasion and metastasis. EMT is also extensively studied in human breast cancer as a biological process contributing to tumor-cell plasticity, invasion, and resistance [33,34]. Nevertheless, the presence of EMT-associated markers in FMC should not be interpreted as proof that identical EMT programs operate in the same manner across species.

### 7.4. Tumor Microenvironment and Immune Regulation

The tumor microenvironment (TME) represents a further area of comparative interest. Studies of FMC have identified tumor-infiltrating lymphocytes and other immune-cell populations, together with alterations in immune checkpoint pathways [16,17,18]. Nascimento et al. characterized the landscape of tumor-infiltrating immune cells in FMC and demonstrated associations between immune-cell composition, pathological characteristics, and clinical behavior [17]. Similarly, PD-1/PD-L1 expression has been investigated in feline mammary carcinoma, including in HER2-positive and triple-negative/normal-like phenotypes [17]. VISTA and TIM-3 have also been proposed as potential immune-regulatory targets in feline mammary carcinoma [18,37].

These observations provide a biological rationale for comparative investigation of tumor–immune interactions. However, the presence of homologous immune checkpoint pathways does not establish that immune checkpoint inhibitors will have equivalent activity in cats and humans. Differences in ligand expression, receptor biology, immune-cell composition, antibody cross-reactivity, pharmacokinetics, and treatment exposure may influence therapeutic responses. Therefore, feline immune checkpoint studies should currently be regarded as hypothesis-generating and complementary to human experimental and clinical research.

The spontaneous development of FMC in immunocompetent hosts may nevertheless provide an opportunity to study tumor–immune interactions under naturally occurring disease conditions [17]. This may be particularly relevant for understanding the relationship between tumor phenotype, immune-cell infiltration, metastatic progression, and treatment response. Prospective studies incorporating standardized pathological, immunological, and clinical endpoints will be necessary to determine the extent to which these findings can be translated across species.

### 7.5. Molecular and Omics-Based Convergence

Recent transcriptomic, proteomic, phosphoproteomic, and peptidomic studies have expanded the molecular characterization of FMC [14,40,41,42]. Transcriptomic profiling has identified candidate biomarkers and druggable pathways related to metabolism and cell-cycle regulation [12], while phosphoproteomic analyses have identified alterations in proteins involved in cell-cycle control, signaling, metabolism, and other processes associated with tumor progression [41]. Tissue proteomic studies have further identified protein signatures associated with metastatic status and histopathological characteristics [40], and serum peptidomic analyses have identified candidate signatures distinguishing metastatic from non-metastatic FMC [42].

These approaches may provide a useful basis for comparative hypothesis generation. For example, several proteins and signaling pathways identified in feline tumors have recognized roles in human breast cancer. However, pathway overlap should be interpreted cautiously. Identification of a conserved protein or signaling pathway does not establish that its regulation, biological importance, or therapeutic response is identical in the two species.

The comparative value of omics approaches will therefore depend on cross-species validation rather than on the identification of isolated molecular similarities. Future studies should ideally compare feline and human tumors using harmonized analytical pipelines, predefined biological questions, sufficiently powered cohorts, and clinically relevant endpoints.

### 7.6. Therapeutic and Translational Relevance

The therapeutic landscape represents an important component of comparative oncology. Surgery remains the principal treatment for FMC, whereas human breast cancer management incorporates surgery, radiotherapy, chemotherapy, endocrine therapy, HER2-directed treatment, immunotherapy, and other targeted approaches according to tumor subtype and stage [5,19]. Systemic treatment studies in cats have generally involved smaller cohorts than those available in human oncology, and evidence for adjuvant systemic treatment remains comparatively limited [19,21,43].

Targeted therapies have nevertheless received increasing attention. Tyrosine kinase inhibitors targeting pathways involving HER2, EGFR, VEGFR, PDGFR, and related receptors have demonstrated antiproliferative activity in feline mammary carcinoma models [20]. Toceranib has also been investigated clinically in cats with macroscopic mammary adenocarcinoma, providing preliminary evidence of biological and clinical activity [21]. These studies illustrate how naturally occurring feline tumors may contribute to comparative therapeutic research.

However, activity of a targeted agent in feline cell lines or clinical patients should not be interpreted as evidence of equivalent efficacy in human breast cancer. Drug pharmacokinetics, target expression, receptor structure, tissue penetration, toxicity, and resistance mechanisms may differ substantially between species. Similarly, evidence that trastuzumab or other HER2-directed approaches interact with feline HER2 in experimental systems supports biological investigation but does not establish clinical equivalence [39].

The most appropriate translational framework is therefore bidirectional and hypothesis-driven. FMC may contribute to the investigation of naturally occurring tumor progression, therapeutic response, resistance, and tumor–host interactions, while findings from human oncology can inform candidate biomarkers and therapeutic strategies for feline disease. This complementary approach avoids treating FMC as a substitute for human breast cancer while recognizing its potential value for selected comparative questions.

### 7.7. Limitations of FMC as a Comparative Model

Several limitations should be considered when interpreting FMC as a comparative model of human breast cancer. First, feline molecular classification remains less standardized than human breast cancer classification. IHC-based surrogate phenotypes are useful for describing biological heterogeneity, but they do not capture the full genomic and transcriptomic diversity of human breast cancer [14,15,47].

Second, methodological heterogeneity remains a major limitation of the feline literature. Differences in tissue processing, antibodies, IHC protocols, scoring criteria, molecular assays, staging systems, and clinical endpoints complicate comparison among studies [5,26]. This problem is particularly evident for HER2 and Ki-67, for which different analytical approaches may generate substantially different results [31,35,49].

Third, many studies of FMC are retrospective and include relatively small cohorts. As emphasized by Zappulli et al., the retrospective nature of much of the available evidence and the lack of methodological standardization reduce the strength of prognostic conclusions and limit comparability between studies [5]. Similar limitations apply to exploratory omics studies, which require validation in independent cohorts before candidate biomarkers can be considered clinically useful.

Fourth, the clinical management of mammary carcinoma differs markedly between cats and humans. Human breast cancer benefits from standardized screening, multidisciplinary staging, prospective clinical trials, and validated systemic therapies. In cats, surgery remains the main therapeutic intervention and evidence supporting systemic and targeted treatments is more limited [19,21,43]. Consequently, treatment response and survival outcomes cannot be directly compared without accounting for these differences.

Finally, the comparative relevance of FMC is unlikely to be uniform across all human breast cancer subtypes. The available evidence suggests particular interest in aggressive, hormone receptor-negative, basal-like, and selected HER2-associated phenotypes [12,14,15,16,17,18]. This does not mean that FMC reproduces the entire biological spectrum of human breast cancer. Instead, its value may be greatest for specific questions concerning aggressive tumor biology, metastatic progression, tumor–immune interactions, biomarker development, and therapeutic response.

Taken together, the available evidence supports the use of FMC as a complementary comparative oncology system rather than as a direct surrogate for human breast cancer. Naturally occurring feline tumors may provide information that is difficult to reproduce in conventional experimental systems, particularly regarding tumor development in an immunocompetent host, spontaneous metastatic progression, and naturally occurring tumor–host interactions. However, translational conclusions should be based on validated molecular and clinical endpoints rather than on phenotypic similarity alone. Standardized pathological and molecular definitions, harmonized methodologies, adequately powered multicenter cohorts, prospective clinical studies, and independent cross-species validation will be required to define the precise translational contribution of FMC to breast cancer research.

**Figure 4:**
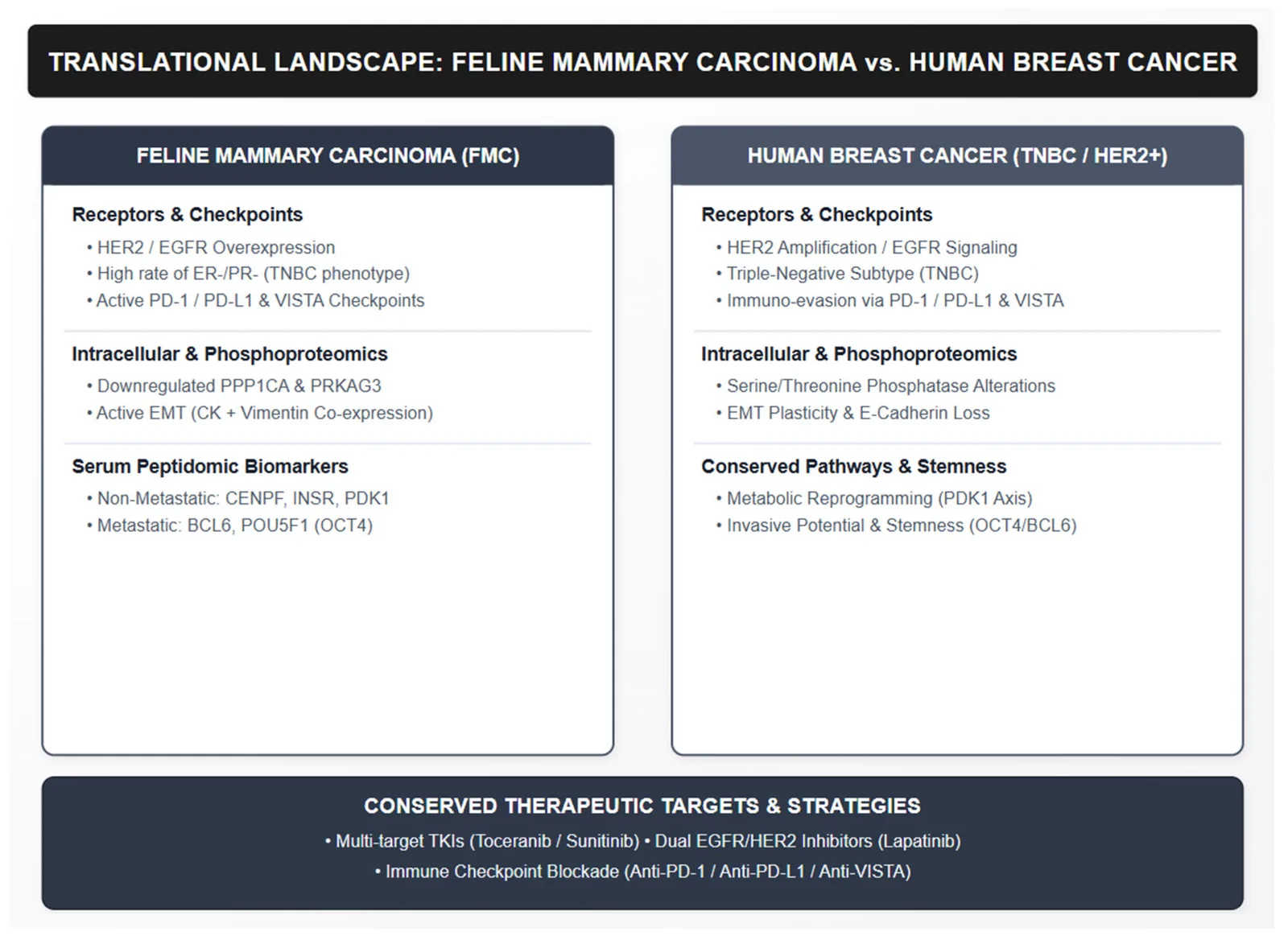
Comparative and translational landscape of feline mammary carcinoma and human breast cancer. The schematic summarizes areas of reported clinicopathological, molecular, immune, and biomarker overlap while emphasizing that these similarities are hypothesis-generating and require cross-species validation.

The main areas of clinicopathological, molecular, immune, and biomarker convergence between feline mammary carcinoma and human breast cancer, together with their principal translational limitations, are summarized in Figure 4 and Table 4.

## 8. Conclusions

Feline mammary carcinoma is an aggressive neoplasm in cats, with frequent local invasion, lymph-node involvement, and distant metastatic progression. Surgery remains the principal treatment, while histopathological grading, clinical staging, and immunohistochemical assessment provide important prognostic information. Recent transcriptomic, proteomic, phosphoproteomic, and serum peptidomic studies have expanded the characterization of FMC and identified candidate biomarkers and signaling pathways. Selected clinicopathological, molecular, and immune features overlap with those described in human breast cancer, particularly in aggressive hormone receptor-negative and selected HER2-associated phenotypes. However, these similarities do not establish biological equivalence. Differences in molecular classification, analytical methods, treatment standards, cohort size, and clinical validation currently limit direct translational inference. FMC is therefore best considered a complementary, question-specific comparative oncology system that may contribute to studies of aggressive tumor biology, metastasis, tumor–host interactions, biomarker discovery, and therapeutic response. Targeted and immunomodulatory approaches remain areas of investigation, but most feline evidence is preliminary. Future studies should prioritize standardized pathological and molecular definitions, harmonized methodologies, adequately powered prospective cohorts, and independent cross-species validation to clarify the specific translational contribution of FMC to human breast cancer research.

## Figures and Tables

**Table 1:** Summary Table of Elston and Ellis classification (EE), mitotic-modified Elston and Ellis classification (MMEE), revised Elston and Ellis (REE) classification based on the histological features of the tumours.

Grading System	Histological Feature	Score
EE, MMEE, REE	1. Tubule formation Present on a high degree >75% of the tumor Moderate degree 10–75% of the tumor Low degree or non-present <10%	1 2 3
EE, MMEE, REE	2. Nuclear pleomorphism Small, regular, uniform nuclei Moderately increased size, vesiculation and variability Vesicular chromatin with variations in size and shape	1 2 3
EE, MMEE, REE	3. Mitotic count EE 0–8 9–16 ≥17 MMEE, REE 0–50 51–70 ≥71	1 2 3 1 2 3
REE	4. Lymphovascular invasion Absent Present	0 1
REE	5. Nuclear form ≤5% abnormal 6–25% abnormal >25% abnormal	1 2 3
**Final score**	**Differentiation**	**Grade**
3–5	Well differentiated	I
6–7	Moderately differentiated	II
8–9 or 8–10 (REE)	Poorly differentiated	III

**Table 2:** Histolopathological classification of feline mammary tumors.

Benignant	Malignant
1. Adenoma Simple Cystadenoma Intraductal adenoma	1. Carcinoma simple Tubular carcinoma Tubulo-papillary carcinoma Cystic-papillary carcinoma Cribriform carcinoma 2. Carcinoma solid 3. Comedocarcinoma 4. Ductal carcinoma 5. Intraductal papillary carcinoma 6. Sarcoma Osteosarcoma 7. Carcinosarcoma

**Table 3:** Clinical staging of feline mammary tumours.

Clinical Stage	Tumour Diameter (T)	Regional Lymph Node (N)	Distant Metastases (M)
I	<2 cm	Negative	Negative
II	2–3 cm	Negative	Negative
III	>3 cm ≤3 cm	Negative or positive Positive	Negative
IV	Any T	Any N	Positive

**Table 4:** Comparative features of feline mammary carcinoma and human breast cancer: evidence, translational relevance, and limitations.

Domain	Feline Mammary Carcinoma	Human Breast Cancer	Comparative Interpretation and Key Limitation
Natural occurrence	FMC occurs spontaneously in cats and frequently presents as an invasive malignant neoplasm [2,3,5,11].	Human breast cancer is a spontaneous disease with a broad spectrum ranging from in situ and indolent lesions to highly aggressive metastatic tumors.	Spontaneous tumor development in immunocompetent hosts is advantageous for comparative oncology, but FMC does not reproduce the complete clinical spectrum of human breast cancer [5,12].
Histopathology	Tubulopapillary, solid, cribriform, micropapillary, and other invasive patterns have been described [5,9,11].	Human breast cancer encompasses a broad WHO-defined spectrum of invasive and in situ entities.	Several morphological patterns overlap, but feline histological categories cannot be directly equated with human breast cancer entities [5,9].
Histological grade	Histological grade and mitotic activity have prognostic value in several studies [7,8,30].	Histological grade is an established prognostic component of human breast cancer assessment.	Biological convergence exists, but grading systems and thresholds are not fully harmonized across species [7,8,26].
Tumor size	Larger tumors are generally associated with more advanced disease and poorer outcome [5,7,8,30].	Tumor size is a major component of TNM staging and treatment planning.	Strong clinicopathological convergence, although staging thresholds and treatment implications differ.
Lymph-node involvement	Regional lymph-node metastasis is associated with advanced disease and poorer prognosis [5,8,15,25].	Regional nodal status is an established component of TNM staging and prognosis.	One of the stronger areas of cross-species clinicopathological convergence.
Distant metastasis	Pulmonary metastasis is common in advanced disease; other distant sites may also be affected [15,25].	Human breast cancer frequently metastasizes to bone, lung, liver, brain, and other organs.	Both species develop spontaneous metastatic disease, but metastatic patterns and clinical management are not identical.
ER/PR expression	Hormone receptor-negative tumors are common, particularly among aggressive carcinomas [5,12,14,15].	ER and PR expression defines major clinical and therapeutic groups of human breast cancer.	FMC may be particularly informative for selected hormone receptor-negative tumors, but feline receptor biology and treatment implications should not be assumed to be identical [12,14].
HER2	HER2 expression has been demonstrated, but reported frequencies vary substantially according to methodology [18,20,49].	HER2 status is clinically standardized and determines eligibility for HER2-directed therapies in appropriate patients [50].	Potentially important conserved pathway, but feline HER2 positivity cannot be directly equated with human HER2-positive disease without standardized validation.
Triple-negative phenotype	ER−/PR−/HER2− FMCs are relatively frequent and have been associated with aggressive clinicopathological features [14,15].	TNBC is defined by absence of ER, PR, and HER2 but represents a heterogeneous molecular group [47].	FMC may represent a phenotypic analogue of selected TNBCs, but IHC receptor negativity alone does not establish molecular equivalence [14,15,48].
Basal-like characteristics	Some feline hormone receptor-negative tumors express basal cytokeratins and vimentin and show aggressive behavior [10].	Basal-like breast cancers represent a molecularly defined group that overlaps substantially, but not completely, with clinical TNBC.	Biologically informative convergence, but feline basal-like tumors should not automatically be assigned to a human molecular subtype [10,47].
Ki-67	Ki-67 has prognostic potential, but reported thresholds and associations vary [31,35].	Ki-67 is used as a proliferation marker and contributes to characterization of selected human breast cancers.	Potentially useful comparative biomarker; however, feline cut-offs should not be extrapolated directly from human studies [5,31,35].
Invasion and EMT	EMT-associated phenotypes and epithelial/mesenchymal marker expression have been investigated in FMC [33,34,41].	EMT-related processes contribute to tumor plasticity, invasion, dissemination, and therapeutic resistance in human breast cancer [33,34].	Shared biological processes support comparative investigation, but individual EMT markers are not validated cross-species prognostic equivalents.
Tumor microenvironment	FMC contains heterogeneous immune and stromal populations, including tumor-infiltrating immune cells [17].	Immune and stromal components are major determinants of breast cancer progression and therapeutic response.	Spontaneous tumors in immunocompetent cats provide a useful setting for investigating tumor–host interactions, but immune composition differs between species [17].
Immune checkpoints	PD-1/PD-L1, VISTA, TIM-3, and CTLA-4-related pathways have been investigated in FMC [16,17,18,37,38].	Immune checkpoints are established therapeutic targets in selected human cancers and breast cancer subtypes.	Shared pathways provide a rationale for comparative investigation; receptor/ligand expression does not demonstrate equivalent therapeutic responsiveness.
Transcriptomics	Transcriptomic studies have identified candidate biomarkers and pathways associated with metabolism and cell-cycle regulation [12].	Large human datasets have established molecular subtypes and clinically relevant genomic signatures.	Feline transcriptomics can generate comparative hypotheses, but cohort size and independent validation remain limiting factors [5,12].
Proteomics/phosphoproteomics	Proteomic and phosphoproteomic studies have identified candidate proteins associated with histological characteristics, metastasis, and signaling [40,41].	Multi-omic approaches are extensively integrated into human breast cancer research.	Potentially valuable for discovery of conserved pathways, but isolated molecular overlap does not demonstrate functional equivalence [40,41].
Liquid biopsy/peptidomics	Serum peptidomic profiles have been reported to distinguish metastatic and non-metastatic FMC [42].	Circulating biomarkers and liquid biopsy approaches are actively investigated in human breast cancer.	Promising comparative biomarker field, but feline findings require larger cohorts and independent validation before clinical application [42].
Surgery	Surgery is the principal treatment for FMC [5,19].	Surgery is integrated with systemic and/or radiation therapy according to stage and molecular subtype.	Major difference in treatment context limits direct comparison of outcomes.
Chemotherapy	Doxorubicin and other cytotoxic protocols have been investigated, with heterogeneous evidence [19,43].	Multiple systemic chemotherapy regimens have been evaluated in large prospective human trials.	Feline evidence base is considerably smaller and less standardized [19,43].
Targeted therapy	HER2-directed approaches and TKIs have shown experimental or preliminary clinical activity [20,21,39].	Multiple targeted therapies have validated indications according to molecular subtype.	FMC can support hypothesis generation and comparative drug research, but feline response cannot substitute for human clinical validation.
Immunotherapy	Immune checkpoint pathways are increasingly characterized, but clinical therapeutic evidence remains limited [16,17,18,37,38].	Immunotherapy has established indications in selected breast cancer settings.	Spontaneous immune-competent tumors are potentially informative, but species-specific immune biology and pharmacology remain important limitations.
Study design	Much of the literature consists of retrospective studies, relatively small cohorts, cell-line experiments, or exploratory omics datasets [5,26].	Human breast cancer research includes large prospective cohorts and randomized clinical trials.	Difference in evidence level substantially limits direct translational inference [5].
Molecular standardization	IHC protocols, antibodies, cut-offs, and molecular classifications vary among studies [5,14,15,49].	Molecular and receptor classification is extensively standardized for clinical use.	Harmonization of feline diagnostic criteria is essential before robust cross-species comparisons can be made [5,26,49].
Overall comparative role	Potentially informative for selected aspects of aggressive mammary tumor biology, metastasis, tumor–immune interactions, biomarkers, and therapeutic response [5,12,17,48].	Reference clinical disease with extensive molecular, pathological, and therapeutic characterization.	FMC should be considered a complementary rather than substitutive comparative model; translational relevance is question-specific and requires independent validation.

## Data Availability

This study was based exclusively on the previously published literature and did not generate or analyze new datasets.

## Supplementary Materials

The following supporting information can be downloaded at https://www.mdpi.com/article/10.3390/cancers18183051/s1. Table S1. Database-specific electronic search strategies used for the integrative review.
