# Supplementary material for: Feline Mammary Carcinoma as a Comparative Model of Human Breast Cancer: An Integrative Review
Source: Cancers (Basel). 2026 Sep 20;18(18):3051. doi: 10.3390/cancers18183051 (PMC13605624; doi:10.3390/cancers18183051)
Supplement: Supplementary file 1 [file cancers-18-03051-s001.zip › cancers-4558575-supplementary.pdf]

Supplementary Table S1. Database-specific electronic search strategies used for the integrative review.

Database/platform	Coverage and last search date	Search fields	Complete search strategy	Filters/limits
**PubMed/MEDLINE**	Database inception to 30 March 2026	Title/Abstract ([tiab])	((("feline mammary carcinoma"[tiab] OR "feline mammary carcinomas"[tiab] OR "feline mammary tumor"[tiab] OR "feline mammary tumors"[tiab] OR "feline mammary tumour"[tiab] OR "feline mammary tumours"[tiab] OR "feline mammary neoplasia"[tiab] OR "feline mammary neoplasm"[tiab] OR "feline mammary neoplasms"[tiab] OR "feline mammary adenocarcinoma"[tiab] OR "feline mammary adenocarcinomas"[tiab])) AND (comparative oncolog*[tiab] OR "human breast cancer"[tiab] OR "human breast cancers"[tiab] OR "breast cancer"[tiab] OR "breast cancers"[tiab] OR "molecular phenotype"[tiab] OR "molecular phenotypes"[tiab] OR "molecular subtype"[tiab] OR "molecular subtypes"[tiab] OR "triple negative"[tiab] OR "triple-negative"[tiab] OR HER2[tiab] OR biomarker*[tiab] OR "tumor microenvironment"[t	No additional restrictions*

			iab] OR "tumour microenvironment"[tiab] OR immunotherap*[tiab] OR proteom*[tiab] OR phosphoproteom*[tiab] OR peptidom*[tiab] OR "tyrosine kinase inhibitor"[tiab] OR "tyrosine kinase inhibitors"[tiab] OR toceranib[tiab])))	
**Scopus**	Database inception to 30 March 2026	Title, abstract and keywords (TITLE-ABS-KEY)	TITLE-ABS-KEY((("feline mammary carcinoma" OR "feline mammary carcinomas" OR "feline mammary tumor" OR "feline mammary tumors" OR "feline mammary tumour" OR "feline mammary tumours" OR "feline mammary neoplasia" OR "feline mammary neoplasm" OR "feline mammary neoplasms" OR "feline mammary adenocarcinoma" OR "feline mammary adenocarcinomas") AND (comparative oncolog* OR "human breast cancer" OR "human breast cancers" OR "breast cancer" OR "breast cancers" OR "molecular phenotype" OR "molecular phenotypes" OR "molecular subtype" OR "molecular subtypes" OR "triple negative" OR "triple-negative" OR HER2 OR biomarker* OR	No additional restrictions*

			"tumor microenvironment" OR "tumour microenvironment" OR immunotherap* OR proteom* OR phosphoproteom* OR peptidom* OR "tyrosine kinase inhibitor" OR "tyrosine kinase inhibitors" OR toceranib)))	
**Web of Science Core Collection**	Database inception to 30 March 2026	Topic (TS=; title, abstract, author keywords and Keywords Plus)	TS=((("feline mammary carcinoma" OR "feline mammary carcinomas" OR "feline mammary tumor" OR "feline mammary tumors" OR "feline mammary tumour" OR "feline mammary tumours" OR "feline mammary neoplasia" OR "feline mammary neoplasm" OR "feline mammary neoplasms" OR "feline mammary adenocarcinoma" OR "feline mammary adenocarcinomas") AND (comparative oncolog* OR "human breast cancer" OR "human breast cancers" OR "breast cancer" OR "breast cancers" OR "molecular phenotype" OR "molecular phenotypes" OR "molecular subtype" OR "molecular subtypes" OR "triple negative" OR "triple-negative" OR HER2 OR biomarker* OR "tumor microenvironment"	No additional restrictions*

			OR "tumour microenvironment" OR immunotherap* OR proteom* OR phosphoproteom* OR peptidom* OR "tyrosine kinase inhibitor" OR "tyrosine kinase inhibitors" OR toceranib)))	

Electronic database searches were complemented by iterative topic-oriented searches and manual screening of the reference lists of key relevant publications and reviews
